# Robot-assisted ureteral reconstruction combined with trans-trocar flexible ureteroscopy using a flexible and navigable suction ureteral access sheath: a case report and technical note

**DOI:** 10.1186/s12894-026-02238-7

**Published:** 2026-07-15

**Authors:** Chaoyue Ji, Jianxing Li, Weiguo Hu, Gang Zhang

**Affiliations:** Department of Urology, Beijing Tsinghua Changgung Hospital School of Clinical Medicine, Tsinghua Medicine, Tsinghua University, 168 Litang Road, Changping District, Beijing, 102218 China

**Keywords:** Ureteral stricture, Robot-assisted surgery, Flexible ureteroscopy, Suction-assisted lithotripsy, Case report

## Abstract

**Background:**

Simultaneous management of upper urinary tract stricture and renal calculi remains a technical challenge in minimally invasive urology. Conventional hybrid techniques combining robotic reconstruction with standard flexible ureteroscopy often face limitations such as impaired visualization, irrigation fluid leakage, and difficulty in managing a substantial stone burden. We report a refined hybrid technique that integrates a flexible and navigable suction ureteral access sheath (FANS) directly into the robot-assisted ureteral reconstructive workflow.

**Case presentation:**

A 49-year-old male patient presenting with a 1-cm middle-to-upper ureteral stricture and a concomitant lower-calyceal stone cluster (aggregate size: 18 × 16 × 12 mm; approximal density: 550 HU) underwent a refined hybrid procedure using the da Vinci Xi system following failed retrograde intrarenal surgery. Intraoperatively, a 12/14 Fr FANS (40 cm) was introduced through an assistant trocar and utilized for 40 min to facilitate thulium fiber laser lithotripsy (1.2 J/10 Hz; active lasing time: 15 min), enabling direct fragment evacuation. Stone-free status was initially confirmed via kidney-ureter-bladder radiography on postoperative day 2. At the 14-month follow-up, abdominal CT demonstrated significant resolution of hydronephrosis and confirmed no recurrence of stones or ureteral obstruction.

**Conclusions:**

The trans-trocar FANS-assisted hybrid approach is a feasible and efficient single-session strategy for complex upper urinary tract pathology. The core innovation lies in the functional integration of suction-assisted endourology into robotic reconstructive surgery.

**Supplementary Information:**

The online version contains supplementary material available at https://doi.org/10.1186/s12894-026-02238-7.

## Background

Robotic platforms have redefined the treatment landscape for ureteral strictures, offering a versatile approach to facilitate the high-precision execution of demanding procedures, such as ureteroureterostomy, Boari flap, and ureteroneocystostomy [1]. However, the management of complex upper urinary tract pathology—characterized by concomitant ureteral strictures and urolithiasis—remains a clinical challenge that typically necessitates a multimodal approach. For such patients, the current ‘state-of-the-art’ often involves staged interventions, such as percutaneous nephrolithotomy (PCNL) followed by delayed robotic repair. Although laparoscopic or robotic reconstruction in combination with trans-trocar flexible ureteroscopy has been reported [2, 3], its applicability is often limited by impaired visualization, irrigation fluid leakage, and difficulty in managing a substantial stone burden. Here, we report a refined hybrid technique that integrates flexible and navigable suction ureteral access sheath (FANS)-assisted ureteroscopy directly into the workflow of robot-assisted ureteral reconstruction, which, to the best of our knowledge, has not been previously described.

## Case presentation

A 49-year-old male presented with left ureteral stricture and ipsilateral renal calculi. He had undergone ureteroscopic lithotripsy for a ureteral stone 18 months earlier at another institution. One month prior to admission, imaging demonstrated left ureteral and renal calculi with a suspected left ureteral stricture. The stricture was estimated to be located distal to the ureteral stone at the level of the lower border of the fourth lumbar vertebra (L4). Renal function was assessed using renal dynamic scintigraphy, which revealed a glomerular filtration rate of 38 mL/min for the left kidney and 64 mL/min for the right kidney. Retrograde intrarenal surgery (RIRS) was initially attempted; however, a severe ureteral stricture was detected distal to the stone, permitting passage of only a guidewire. Subsequent balloon dilation (4 mm in diameter, 40 mm in length; INNOVEX, Shanghai, China) under direct vision failed to facilitate the insertion of a 10/12 Fr ureteral access sheath. The procedure was terminated after placement of two double-J stents.

**Fig. 1:**
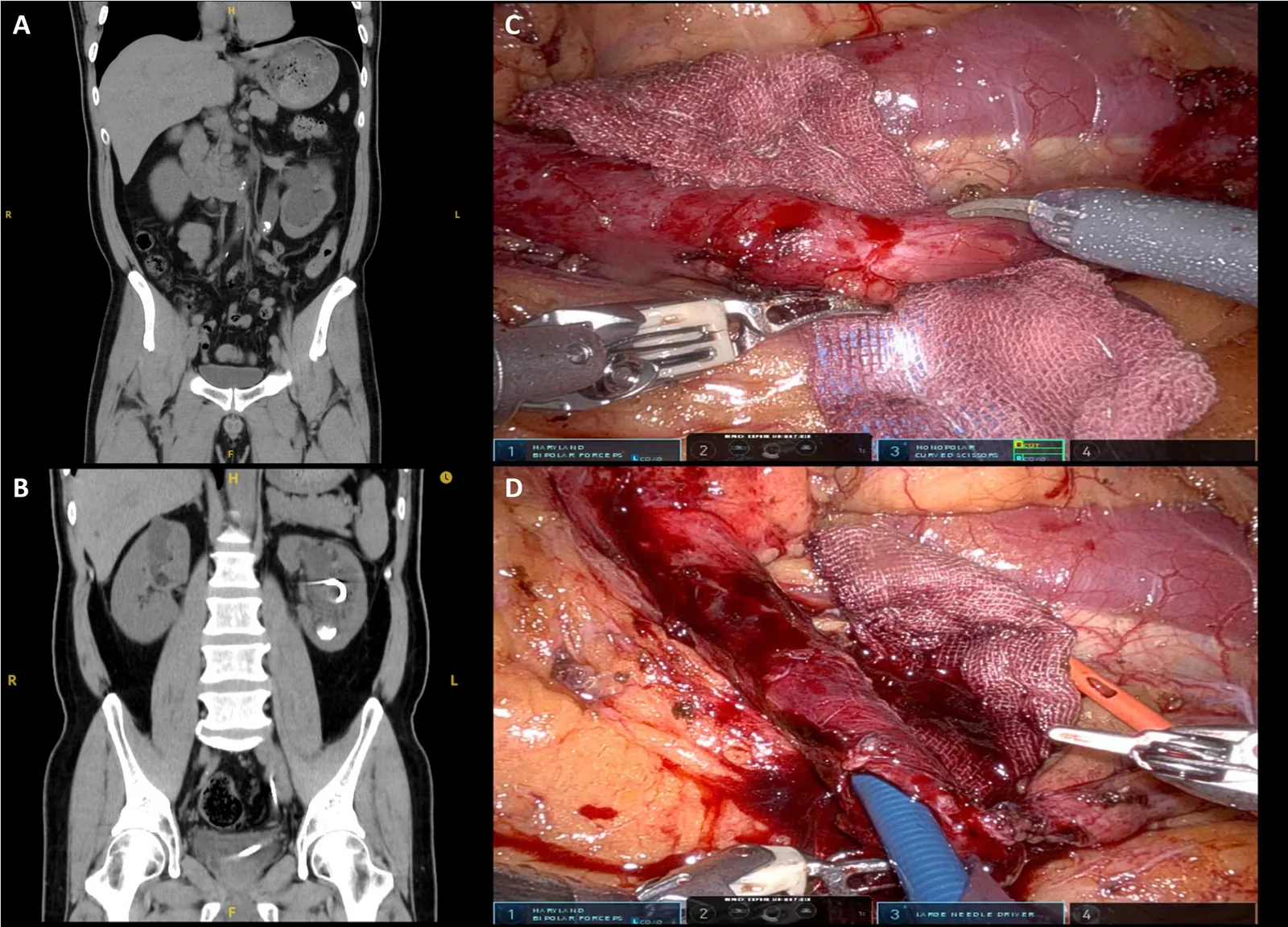
**A** Preoperative non-contrast abdominal computed tomography showing the site of ureteral obstruction prior to transurethral flexible ureteroscopic lithotripsy. **B** Preoperative non-contrast abdominal computed tomography revealing a substantial renal stone burden predominantly located in the lower calyx, supporting the feasibility of suction-assisted stone management before robot-assisted ureteral reconstruction. **C** Intraoperative robotic view revealing the localized ureteral stricture segment. **D** Intraoperative robotic view illustrating trans-trocar insertion of a flexible and navigable suction sheath through ureterotomy proximal to the stricture, enabling simultaneous flexible ureteroscopic lithotripsy and stone extraction

Following a comprehensive evaluation, a transperitoneal robot-assisted laparoscopic ureteral stricture resection and ureteroureterostomy were planned and subsequently performed. The preoperative imaging findings and key intraoperative steps are summarized in Fig. 1. Non-contrast abdominal computed tomography (CT) revealed a cluster of multiple calculi in the lower calyx. The aggregate maximal dimensions of this stone cluster were 18 × 16 × 12 mm, with an estimated total volume of approximately 1,809mm^3^ (calculated using the ellipsoidal formula: 0.523×L×W×H). The mean stone density was 550 Hounsfield units. Two separate preoperative urine cultures were performed prior to the robotic reconstruction, both of which yielded negative results.

The patient was placed in a 70-degree lateral decubitus position under general anesthesia. All procedures were carried out using the da Vinci Xi Surgical System (Intuitive Surgical, Sunnyvale, CA, USA). Following the establishment of pneumoperitoneum at 12 mmHg, the trocar configuration was optimized for a left-sided approach. Three 8-mm robotic trocars (Arms 1, 2, and 3) were placed linearly along the outer edge of the left rectus abdominis muscle with an inter-port distance of 8–10 cm. Specifically, the camera port (Arm 2) was equipped with a 30° robotic endoscope and positioned at the level of the umbilicus. The cranial port (Arm 1) was used for Maryland bipolar forceps, while the caudal port (Arm 3) was utilized for monopolar curved scissors. A 12-mm assistant port, serving as the dedicated entry point for the FANS, was placed slightly superior and left lateral to the umbilicus to facilitate seamless instrument coordination. Intraoperatively, a localized stricture approximately 1 cm in length was identified at the middle-to-upper ureter. After ureterotomy proximal to the stricture, a 7.5 Fr disposable digital flexible ureteroscope (Zebrascope, Anhui, China) was initially introduced through the incision to inspect the collecting system. A cluster of stone fragments was observed in the lower calyx, characterized by a substantial stone burden and a purulent coating. Subsequently, a 40 cm, 12/14 Fr FANS (INNOVEX, Shanghai, China) was introduced through the robotic trocar using a 0.035-inch guidewire (INNOVEX, Shanghai, China). Irrigation was maintained using a constant-pressure irrigation pump set at 500mmH_2_O, while suction was provided by a continuous wall-mounted suction system. The FANS was utilized throughout the endourological phase (approximately 40 min), with active lasing time totaling 15 min. Laser lithotripsy was performed using a domestically manufactured super-pulse thulium fiber laser (A-one, Beijing, China) with parameters set at 1.2 J and 10 Hz in short-pulse mode. FANS-assisted flexible ureteroscopic lithotripsy enabled efficient stone fragmentation and direct evacuation of debris, achieving complete stone clearance in a single session. Ureteral reconstruction was then completed in a standard fashion. The total operative time was 234 min, with minimal intraoperative blood loss.

The postoperative course was uneventful. On postoperative day (POD) 1, the white blood cell count showed a minor elevation (11.5 × 10⁹/L) but normalized by POD 3. Procalcitonin levels remained within the normal reference range throughout the recovery period. Additionally, both hemoglobin and serum creatinine levels remained stable, with no significant postoperative fluctuations compared to preoperative values. Kidney-ureter-bladder radiography (KUB) on postoperative day 2, as per our institutional protocol, indicated a stone-free status by KUB criteria, acknowledging the inherent limitations of plain radiography in detecting tiny or radiolucent fragments. Stone analysis revealed calcium oxalate monohydrate and carbonate apatite. At the 14-month last follow-up, a repeat non-contrast abdominal CT was performed, which served as the definitive confirmatory assessment, demonstrating significant relief of the left hydronephrosis and confirming no recurrence of renal or ureteral calculi. No complications were observed during the entire follow-up period.

### Discussion and conclusions

Previous reports have described simultaneous laparoscopic or robotic pyeloplasty in combination with trans-trocar flexible ureteroscopy for stone removal, demonstrating the feasibility of endoscopic-assisted robotic reconstruction in selected patients [2, 3]. These approaches offer advantages such as completion in a single position, avoidance of renal parenchymal injury, and no need for additional incisions. Conventional flexible ureteroscopy through an open collecting system is often hampered by irrigation fluid leakage, suboptimal visualization, and the risk of stone fragment spillage into the peritoneal cavity, particularly in cases with heavy stone burden or infected calculi.

FANS-assisted flexible ureteroscopy has been shown to maintain a clear operative field, reduce intrarenal pressure and infectious complications, minimize thermal injury, and improve stone-free rates [4–8]. Our approach builds upon established hybrid methods by specifically integrating FANS technology. The primary technical distinction lies in the application of active suction, which enhances the existing surgical paradigm and addresses the aforementioned limitations of conventional endourology in laparoscopic/robotic system. Firstly, and most significantly, FANS may improve the feasibility of managing a substantial stone burden during reconstruction with superior clearance efficiency, thereby avoiding staged procedures or percutaneous access and preserving the renal parenchyma. Secondly, the continuous balance of irrigation and suction stabilizes intraoperative visualization. Thirdly, the active suction allows for the direct evacuation of stone fragments, thereby minimizing the risk of intraperitoneal dissemination. To mitigate peri-FANS leakage at the ureterotomy site, we balanced the inflow and outflow by dynamically adjusting the wall suction intensity against a constant irrigation flow rate. This approach effectively maintained low intrarenal pressure and minimized fluid escape into the abdominal cavity. Any minor residual leakage was easily cleared using a suction device through the assistant trocar following the completion of the endoscopic procedure. Nevertheless, while the successful management of this complex case underscores the potential of the trans-trocar FANS-assisted hybrid technique, it is important to acknowledge the inherent limitations of a single-case experience. This report primarily represents a preliminary feasibility demonstration rather than a validated clinical approach.

This hybrid approach is not without challenges. The technical difficulty lies in the requirement that surgeons must be proficient in both robotic reconstruction and FANS-assisted ureteroscopy to ensure seamless transitions between ureterotomy, lithotripsy, and precise anastomosis as well as appropriate selection of devices and parameter settings. Trocar placement must be carefully planned to prevent instrument collision, while permitting robotic assistance during sheath insertion. To mitigate port angulation during guidewire-led FANS insertion, the assistant must manually adjust the orientation of the assistant trocar toward the ureterotomy. Simultaneously, the surgeon utilizes the robotic Maryland bipolar forceps to lift and orient the proximal ureteral stump, creating a more favorable angle for smooth sheath advancement. During lithotripsy, the position of the FANS is manually stabilized and adjusted in a manner similar to standard RIRS techniques; in our experience, no sheath slippage or displacement from the ureterotomy occurred.

Despite these technical complexities, the 14-month follow-up in this case, characterized by resolved hydronephrosis and the absence of complications or recurrence, supports the mid-to-long-term feasibility and durability of this FANS-assisted robotic hybrid approach. Its clinical applicability can be further optimized through strategic patient selection. Ideal candidates are those with concomitant middle-to-upper ureteral strictures or ureteropelvic junction obstruction accompanied by a significant stone burden in the renal pelvis or calyces. Conversely, in cases of lower ureteral strictures, the acute angulation required for sheath insertion via the ureterotomy may hinder endoscopic maneuverability. This refined technique is particularly beneficial in scenarios where conventional staged procedures (e.g., antegrade PCNL followed by delayed reconstruction) pose elevated risks of hemorrhage or parenchymal injury—especially in high-risk surgical candidates, patients with solitary kidneys, or those with complex anatomical variations.

In conclusion, robot-assisted ureteral reconstruction in combination with trans-trocar FANS-assisted flexible ureteroscopic lithotripsy represents a feasible single-session strategy for complex upper urinary tract pathology. The core innovation lies in the functional integration of suction-assisted endourology into robotic reconstructive surgery. Moving forward, the integration of minimally invasive platforms is expected to evolve from the manual combination of separate devices toward AI-driven, all-in-one intelligent systems, further minimizing surgical trauma and optimizing clinical outcomes. Regarding the currently explored hybrid techniques, further multi-center studies with larger cohorts are required to confirm their long-term outcomes and generalizability.

## Supplementary Information

Supplementary Material 1.

## Data Availability

The datasets used and/or analyzed during the current study are available from the corresponding author on reasonable request.

## Abbreviations

FANS: Flexible and navigable suction ureteral access sheath
PCNL: Percutaneous nephrolithotomy
RIRS: Retrograde intrarenal surgery
CT: Computed tomography
POD: Postoperative day
KUB: Kidney-ureter-bladder radiography
